# National hepatitis registry in Pakistan: a dire need for hepatitis surveillance and control

**DOI:** 10.1186/s41182-023-00534-8

**Published:** 2023-08-04

**Authors:** Muhammad Farhan, Faizan Fazal, Tirth Dave, Armeen Butt, Jawad Basit, Shahzaib Maqbool

**Affiliations:** 1 Rawalpindi Medical University, Rawalpindi, Pakistan; 2 Bukovinian State Medical University, Chernivtsi, Ukraine; 3 Benazir Bhutto Hospital, Rawalpindi, Pakistan

**Keywords:** Hepatitis B, Hepatitis C, HBV, HCV, National registry, National database

## Abstract

Hepatitis is a major public health issue in Pakistan, with an estimated 11.55% prevalence of HCV infection in the adult population. The country ranks second globally in terms of hepatitis C virus (HCV) infections, with approximately one in every 20 Pakistanis already infected. The mortality rates due to HBV and HCV stand at 563,000 and 366,000 annually, respectively. However, the absence of a national registry or database system and the lack of coordination among provinces pose significant obstacles in combating this disease effectively. To address this issue, the establishment of a centralized national database registry is crucial, allowing comprehensive analysis, tracking of hepatitis prevalence, and identification of high-risk areas for targeted interventions. By fostering collaboration among provinces, the government, and non-governmental organizations, the registry would facilitate joint decision-making, minimize duplication of efforts, and address inconsistencies in diagnosis and treatment. Collaborating with student-run organizations and leveraging enhanced laboratory capacities post-COVID era can strengthen the hepatitis control program. The centralized approach and unified efforts are necessary to achieve the goal of a hepatitis-free Pakistan, where a healthier future can be realized.

**Dear Editor**

Hepatitis is a major public health issue in Pakistan. Globally more than 2 billion people are reported to have been infected with hepatitis. HBV infection has the highest share followed by infection with HCV resulting in an annual death rate of around 1.4 million from hepatitis-related complications [1]. With one in every 20 Pakistanis already infected, Pakistan has the second largest number of hepatitis C virus (HCV) infections globally [2]. A review of ninety different studies conducted in Pakistan observed that the prevalence of HCV infection in the adult population of Pakistan was 11.55%. Drug users were found to have the highest prevalence of 51%. The percentage prevalence of HCV found for all of the provinces was Punjab: 5.46%, Sindh: 2.55%, Khyber Pakhtunkhwa: 6.07%, Baluchistan: 25.77%, and federally administrated tribal areas: 3.37% (Fig. 1). The study further observed that the average prevalence in the whole of Pakistan was a whopping 8.64% [3]. According to the WHO report on prevention and control of hepatitis [4] it has been mentioned that in Pakistan almost 12 million people are suffering from hepatitis B or C. The funds allocated by the Pakistani government for hepatitis control and treatment have unfortunately not yielded the expected results. To reach the HVC Elimination targets set by WHO, Pakistan would require to have an annual average of 18.8 million screens, 1.1 million treatments, and 46,700 new infections prevented annually between 2022 and 2030 [5]. The country’s annual death rate from Hepatitis is still on the rise. The mortality due to HBV is 563,000 annually whereas, the mortality due to HCV is 366,000 annually [6].

**Fig. 1:**
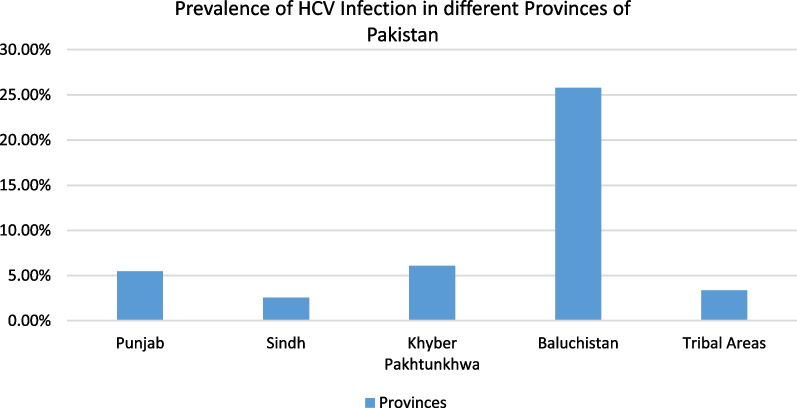
Prevalence of HCV infection in provinces of Pakistan [3]

One of the reasons for the above-mentioned worrisome statistics is the absence of a national registry or database system. High prevalence of HBV and HCV in various provinces of Pakistan as described in the first paragraph is indeed a worrisome statistic. National registers and databases can help in assessing the current status of any disease in a country. Multiple countries in the globe like Denmark are already getting benefits from this initiative and they update the prevalence and effectiveness of their programs using these registries [7]. Similar system of a singular, comprehensive, and inclusive national registry is required that can keep tract of the statistics related to HBV and HCV on the aspects of prevalence, treatment, and follow up of these patients. Currently, each province has its own program, such as the Punjab Hepatitis Control Program, Hepatitis Free Sindh Program, etc., resulting in a lack of uniform policies for screening, testing, and data management, and these programs are supervised by a federal technical advisory group. This lack of coordination and unified approach across provinces presents significant obstacles in effectively combating this disease.

To address this issue and achieve effective control of hepatitis in Pakistan, it is crucial to urgently take steps in establishing a centralized national database registry. This registry would collect data from across the country, enabling comprehensive analysis to track the prevalence of hepatitis and identify high-risk areas guiding in micro elimination. By doing so, proper resource allocation and develop uniform policies and guidelines for all federating units can be ensured. This registry would enhance the consistency of approaches and improve the effectiveness of hepatitis control programs. No national study has been conducted to report the accurate prevalence of hepatitis in Pakistan since 2008 and our programs are based on provincial data which is a passive surveillance system with a lot of loopholes. We need national data based on an active surveillance system. The establishment of a national database registry would foster collaboration among provinces, the federal government, and non-governmental organizations. It would facilitate joint decision-making based on the exchange of information, minimizing duplication of efforts, and addressing inconsistencies in the diagnosis and treatment of hepatitis. A targeted intervention, supported by accurate national data, is the only viable solution to combat this endemic disease. This centralized approach will eradicate the disparities in case definitions. Without a centralized approach, it will be challenging to achieve the goal of a hepatitis-free Pakistan.

One of the options to estimate the prevalence of HBV and HCV in Pakistan is to integrate the assessment of HBV and HCV in the national population survey that occurs after every few years to estimate the country’s total population and other demographic characteristics. By doing this it would be ensured that the whole population is assessed rather than covering only a limited regional population. But to screen for HBV and HCV at such large scale would require a lot of equipment and trained manpower. But this seems worth it considering the staggering statistics of morbidity and mortality that is associated with HBV and HCV infection in Pakistan.

Another option to assess the prevalence of HBV and HCV in Pakistan is to setup HBV and HCV screening centers in each union council of Pakistan. The whole country is divided into union council to be governed by local bodies. The presence of clearly demarcated and functional union council system of Pakistan can be used for the benefit of assessing the prevalence of HBV and HCV by setting state-of-the-art screening centers in each of the union council. This will have the huge advantage of being all inclusive. Another advantage it would provide is that it will give statistics related to prevalence of HBV and HCV in each separate union council and thus it will be easy to analyze the areas where the prevalence is more and resources can then be diverted to that specific area. But this too will require a lot of resources and proper campaigning to ensure that people feel it necessary and motivated to be screened.

Out of both options, the option of setting HBV and HCV screening centers in each union council of Pakistan seems to be more feasible. These centers will basically serve as a diagnostic center for HBV and HCV. The centers can have trained professionals for screening of HBV and HCV and for the data entry. The data from each center can then be evaluated to assess the overall prevalence of HBV and HCV in the whole country and in each province. Later, these HBV and HCV screening centers can be upgraded to include other diagnostic facilities for other chronic diseases as well. These screening centers would also be available to serve any additional health duties in times of pandemics and endemics. Thus, the option of having these screening centers is quite beneficial and the need of time. Initially developed to assess the prevalence of HBV and HCV in the country, these centers can play a bigger role in the long term by assuring that people get important diagnostic facilities which are affordable to the people.

These screening centers established in each union council then can contribute their data to the national hepatitis registry. And thus, the national hepatitis registry will have the data from all over Pakistan regarding the prevalence of HBV and HCV. The data of patients found to be positive for HBV and HCV can then be added to the national hepatitis registry at those screening centers at each union council of Pakistan. This will not only help to assess the prevalence of HBV and HCV, but it will also help in assessing the demographic variables associated with hepatitis. This will help to find out any factors that are contributing to increased hepatitis infection in a certain union council. The national hepatitis registry will also help to assess the improvement in statistics of hepatitis after doing certain interventions to control HBV and HCV. Thus, it will help in determining which interventions are useful to control and eliminate hepatitis. Moreover, setting up screening centers and connecting each screening center with a single national hepatitis registry will help in directing the useful resources in those areas where hepatitis prevalence is found to be high relative to other areas. This can only be done by setting screening centers in union councils and then connecting those with a single national hepatitis registry.

The government of Pakistan can join hands with nongovernment organizations including student-run bodies in medical universities and colleges for the collection of data and screening purposes as many student-run organizations like Volunteer Force against Hepatitis Transmission (VFAHT) are already actively conducting screening camps for the general masses. We can replicate the models of the Rawalpindi Hepatitis Free initiative and Rawalpindi Medical University Hepatitis Free zone initiative with further improvements on a national scale to utilize the trained human resource comprising students and health professionals for screening, data collection, and research purposes [8]. By tapping the human resource available in our educational institutes, we can enhance the reach and impact of our hepatitis control program with minimal financial burden. Furthermore, it will strengthen the existing infrastructure of volunteer bodies of students and foster a sense of ownership among the young health professional toward public health issues. In the post-COVID era, we have an upgraded infrastructure with enhanced capacities of our laboratories. Our national capacity to conduct PCR tests and screening has significantly increased because of resource allocations during the COVID era. So we can use this to our advantage to deal with hepatitis issues.

In conclusion, it is imperative to recognize the pressing need for a centralized national database registry in order to effectively control hepatitis in Pakistan. By rectifying the fragmented and disintegrated approach currently in place, we can work towards a future where our endeavors against hepatitis are unified, well-coordinated, and ultimately achieve success in creating a healthier Pakistan.

## Data Availability

Not applicable.
